# Studying the Effect of Reducing Agents on the Properties of Gold Nanoparticles and Their Integration into Hyaluronic Acid Hydrogels

**DOI:** 10.3390/molecules29245837

**Published:** 2024-12-11

**Authors:** Elżbieta Adamska, Agata Kowalska, Anna Wcisło, Katarzyna Zima, Beata Grobelna

**Affiliations:** 1 Faculty of Chemistry, University of Gdansk, Wita Stwosza 63, 80-308 Gdansk, Poland; agata.kowalska@ug.edu.pl (A.K.); anna.wcislo@ug.edu.pl (A.W.); 2 Department of Physiology, Faculty of Medicine, Medical University of Gdansk, Debinki 1, 80-210 Gdansk, Poland; katarzyna.zima@gumed.edu.pl

**Keywords:** hydrogel masks, hyaluronic acid, nanogold, permeability, biological membranes, pig skin

## Abstract

Gold nanoparticles (Au NPs) are a promising target for research due to their small size and the resulting plasmonic properties, which depend, among other things, on the chosen reducer. This is important because removing excess substrate from the reaction mixture is problematic. However, Au NPs are an excellent component of various materials, enriching them with their unique features. One example is hydrogels, which provide a good, easily modifiable base for multiple applications such as cosmetics. For this purpose, various compounds, including hyaluronic acid (HA) and its derivatives, are distinguished by their high water-binding capacity and many characteristics resulting from their natural origin in organisms, including biocompatibility, biodegradability, and tissue regeneration. In this work Au NPs were synthesized using a green chemistry method, either by using onion extract as a reductant or chemically reducing them with sodium citrate. A complete characterization of the nanoparticles was carried out using the following methods: Fourier-Transform Infrared Spectroscopy (FT-IR), Electrophoretic (ELS), and Dynamic Light Scattering (DLS) as well as Transmission Electron Microscope (TEM) and Scanning Electron Microscope (SEM). Their antioxidant activity was also tested using the 2,2-diphenyl-1-picrylhydrazyl radical (DPPH). The results showed that the synthesized nanoparticles enrich the hydrogels with antioxidant properties and new surface properties (depending on the reducing agent, they can be more hydrophilic or hydrophobic). Preliminary observations indicated low cytotoxicity of the nanomaterials in both liquid form and as a hydrogel component, as well as their lack of penetration through pig skin. The cosmetic properties of hydrogel masks were also confirmed, such as increasing skin hydration.

## 1. Introduction

Today, sustainability, the development of methods of green chemistry, and environmental protection are objects of interest for many branches of science and industry. Environmentally friendly, biodegradable cosmetics are becoming popular. As a result, the use of nano-sized materials is gaining increasing attention, and they have the potential to be used not only in the cosmetic industry but also in the food, medical, and pharmaceutical industries [1,2].

In the cosmetics industry, nano-sized ingredients are often added because formulations containing nanometric particles have more desirable properties. One of the most interesting nano-scale raw materials is nanogold (Au NPs), which can be tailored to provide a variety of functions and applications [3,4]. They can be functionalized by attaching a variety of ligands, biomolecules, or polymers to the nanoparticle surface. Au NPs have broad applications in biomedical fields such as medical diagnostics [5,6,7,8], cancer treatment [9,10,11], drug delivery [12,13], and bioimaging [14,15,16,17]. Compared to larger gold particles, they exhibit enhanced surface activity, chemical reactivity, and modified optical properties. Furthermore, Au NPs are highly biocompatible, making them well-tolerated by living organisms. Unfortunately, commercial products containing nanomaterials have become a source of nanoparticles, which are released into ecosystems and lead to their accumulation in living organisms [18]. In addition, conventional methods of NPs synthesis involve chemical reduction. The size of nanoparticles strongly depends, among other things, on the chosen reducer and stabilizers. Many of these chemicals pose risks to the environment [19]. Therefore, green chemistry principles have become a focus of nanoscience research. Numerous studies have highlighted using natural compounds or sources in the green synthesis of Au NPs [20].

Hyaluronic acid (HA) is an anionic polysaccharide consisting of D-glucuronic acid and N-acetylglucosamine. HA is highly hydrophilic and can absorb large amounts of water through hydrogen bonds, forming a viscous and flexible gel. In an aqueous solution, the molecule of this acid is stabilized by hydrogen bonds parallel to the chain axis. Consequently, it adopts a stiffened helix configuration, giving the molecule an extended coiled-coil structure [21,22]. Due to its biocompatibility and biodegradability, HA and its derivatives are being extensively studied for biomedical applications such as drug delivery, tissue engineering, and molecular imaging. The biocompatibility of HA is due to its natural occurrence in the body and its non-immunogenic nature. This means it is unlikely to provoke an immune response in the body, reducing the risk of rejection. Its biodegradability, in turn, means that it can be broken down and metabolized by the body over time. This is due to specific enzymes in the body, such as hyaluronidases, which can cleave the glycosidic bonds between the disaccharide units of HA. This process can be controlled by modifying the polymer chains’ molecular weight and degree of cross-linking. In addition, HA can bind to cell surface receptors, such as CD44 and RHAMM, expressed on osteoblasts and osteoclasts [21,23].

One of the important properties of HA in developing new materials is its ease of modification due to its chemical structure containing various polymeric side groups, such as hydroxyl, acetamide, and carboxyl groups. Thanks to this, functional groups can be introduced into its structure, which is used to cross-link and incorporate bioactive molecules. This allows the creation of HA-based hydrogels with tailored physical and chemical properties, such as degradation rate, mechanical strength, and bioactivity [24]. Various materials are used to functionalize such hydrogels, including peptides [25,26], nucleotides [27], ions, synthetic and natural polymers [28,29,30], or nanoparticles [31,32]. The latter are of great interest due to their sizeable active surface and various properties resulting from their plasmonic nature. One of the most interesting examples is Au NPs, which are known for their beneficial effects on wound healing [33,34,35]. They are known for their anti-inflammatory and antioxidant properties, as well as their impact on collagen formation, which leads to faster wound recovery.

The research aimed to create a multifunctional material by combining the properties of the hydrogel itself and the properties of nanoparticles obtained using two different reducing agents and to conduct basic research, which will be the basis for further work and development of this research direction and more advanced research. A significant difference in these materials is the use of a natural reducer in the name of the principles of green chemistry, which has a beneficial effect on the environment and does not contain hazardous substances.

Onion-based Au NPs, for the first time, were incorporated into HA-based hydrogels to obtain new nanocomposites for medicinal or cosmetics applications. Our studies show that bridging the gap between sustainable production methods and advanced nanostructure designs is possible. Thanks to this, we can develop next-generation nanomaterials that are both environmentally friendly and highly functional, paving the way for new advancements in biomedicine, cosmetology, and environmental science.

## 2. Results and Discussion

### 2.1. The Phytochemical Study of Onion Allium cepa L. Extract

**Figure 1:**
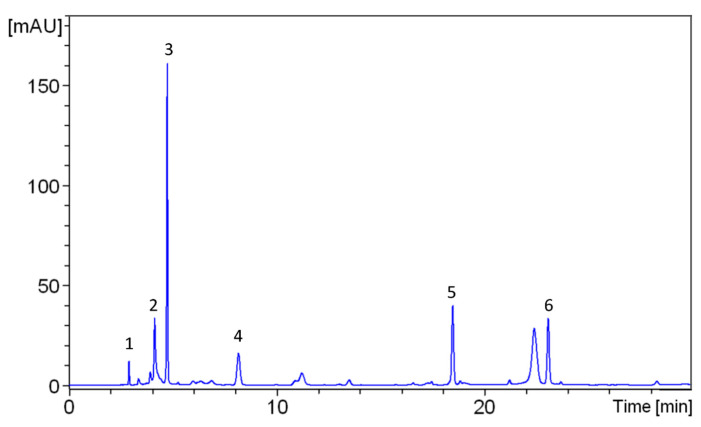
HPLC-UV chromatograms of onion extract acquired at 254 nm. Peak identification: 1—Gallic acid, 2—Myricetin, 3—Catechin, 4—Caffeic acid, 5—Quercetin, 6—Rutin.

A phytochemical analysis was conducted to investigate the presence of organic compounds in the extracts used. To further demonstrate the presence of polyphenols, quantitative High-Performance Liquid Chromatography-Ultraviolet Detection (HPLC-UV) analysis of phenolic acids and flavonols in the extract was performed. According to the literature, the onion extract was analyzed using this method, which was previously optimized to determine the main polyphenols [36,37]. Typical HPLC-UV chromatograms of the extract are shown in Figure 1. From a qualitative point of view, the onion extracts contained almost exclusively phenolic acids and flavonols; in particular, the most abundant compounds were catechin and quercetin.

### 2.2. Characterization of Obtained Nanostructures

**Figure 2:**
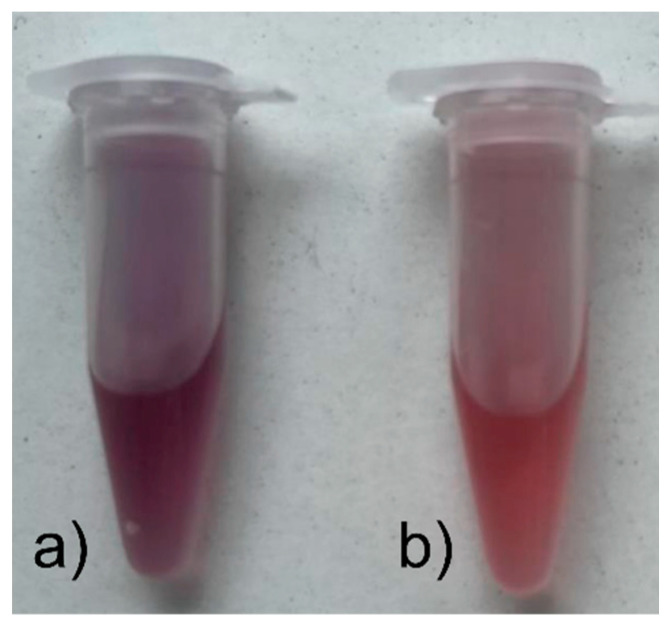
Synthesized colloids of Au NPs obtained with the reducer (**a**) onion extract; (**b**) sodium citrate.

The resulting Au NPs colloids were different in color. Those made with onion extract as a reductant were purple, while those with sodium citrate were ruby-colored (Figure 2).

**Figure 3:**
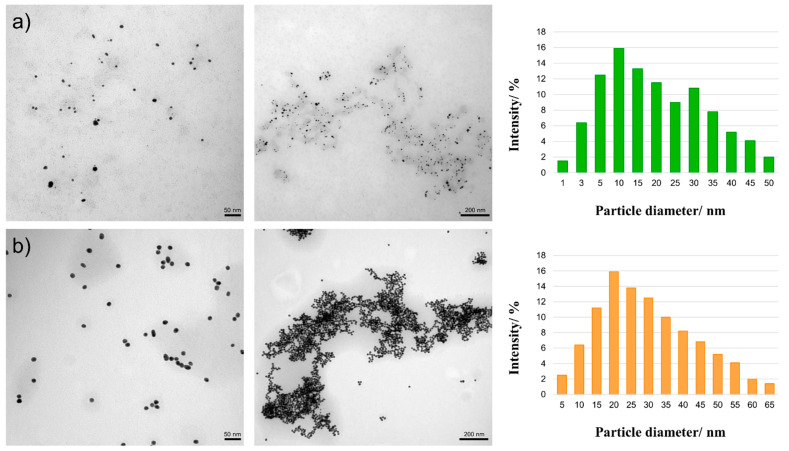
TEM images and diagrams of the particle diameter for Au NPs obtained using the reducer (**a**) onion extract; (**b**) sodium citrate on two scales: 50 and 200 nm.

The basic technique for determining the shape, size, and surroundings of nanoparticles is transmission electron microscopy (TEM). Both obtained nanoparticles are spherical. In the case of nanoparticles obtained using onion extract (Figure 3a), the diameter of the nanoparticles is about 10–30 nm; in the case of those using sodium citrate (Figure 3b), it was 20 nm. In the second case, the size and shape are more uniform, due to the nature of the chemical-reducing agent. Natural reducing agents are most often multicomponent mixtures; therefore, it can be difficult to control various parameters of the nanostructures.

The size of the obtained nanoparticles also plays an important role, among others, considering the possible later applications of nanomaterials in various industrial fields such as cosmetology or medicine. The detailed results of particle size measurements performed by the DLS method of the prepared nanoparticles are shown in Table 1. The nanoparticles obtained using sodium citrate are quite uniform, and the average size is about 67 nm. On the other hand, the result for the nanoparticles obtained using onion extract showed two sizes of 24 nm and about 199 nm, which means that they are more diverse. This may also be due to the measurement of clusters of small nanoparticles into one larger agglomerate.

The polydispersity index (PdI) measures sample heterogeneity based on size. When its value does not exceed 0.1, the system is monodispersed, and above this value, it is polydispersed [38]. This index was 0.23 for nanoparticles obtained using onion extract and 0.08 for nanoparticles obtained using sodium citrate. These results illustrate the potential advantage of the chemical reducer in obtaining a more uniform size in contrast to the applied reducer used in the green chemistry method.

Zeta potential is a variable used to measure the surface charge of NPs and informs about the stability of nanoparticle suspensions. Generally, colloids where the zeta potential remains greater than +25 or less than −25 mV are considered stable suspensions.

In the case of nanoparticles obtained using onion extract, zeta potential values were stable (Table 2). In turn, the potential value differs slightly from the expected values for stable structures for nanoparticles synthesized using sodium citrate. However, this is a slight deviation from the norm, which may be caused, for example, by significant Brownian movements in the solution.

**Figure 4:**
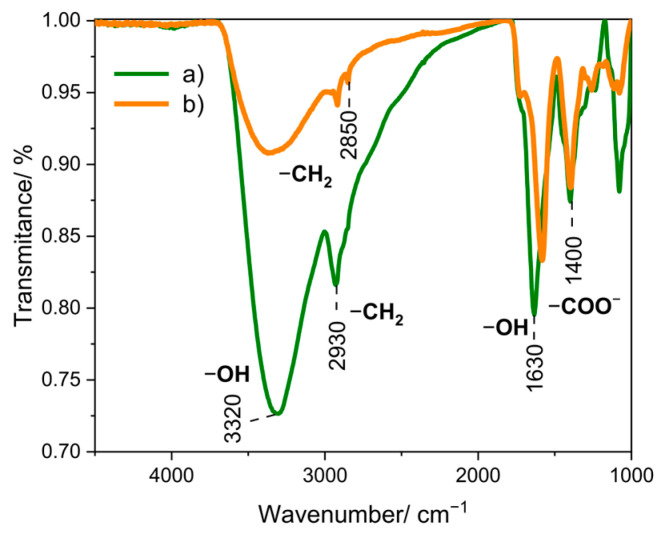
FT-IR spectra for Au NPs obtained using the reducer (**a**) onion extract, and (**b**) sodium citrate.

Fourier-transform infrared spectroscopy (FT-IR) is an analytical technique used to study the structures of molecules and identify the functional groups of a chemical compound. On the FT-IR spectrum for Au NPs synthesized using sodium citrate (Figure 4b), characteristic bands at 3360 cm^−1^ are noticeable, which corresponds to stretching and bending vibrations of the −OH groups, and bands 2920 and 2850 cm^−1^, which can be attributed to stretching vibrations in the −CH_2_ groups [39]. The 1590 and 1400 cm^−1^ bands are assigned to asymmetric and symmetric stretching vibrations, respectively, resulting from the presence of −COO^−^ groups. This is related to the presence of carboxyl groups in sodium citrate [40]. In the case of Au NPs synthesized using onion extract (Figure 4a), a band located at 3320 cm^−1^ is visible, corresponding to the stretching and bending vibrations of the −OH groups. In addition, a band located at 2930 cm^−1^ is assigned to the (CH) modes of the −CH_2_ group [41]. The band observed at 1400 cm^−1^ may correspond to the −CH group found in alkenes [42]. This is due to the presence of many flavonoids in the onion extract, in the structure of which the pyran ring occurs [43].

### 2.3. Antioxidant Properties

**Figure 5:**
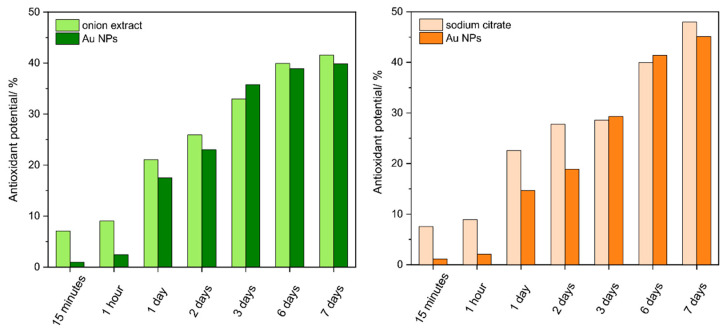
DPPH radical scavenging activity of reducer and Au NPs obtained using onion extract ((**left**) diagram) and sodium citrate ((**right**) diagram) after 15 min, 1 h, and 1, 2, 3, 6, 7 days. Results are expressed in terms of mean ± SEM (*n* = 3).

The antioxidant activity of the obtained Au NPs and the corresponding reductants was assessed by the percentage inhibition of 2,2-diphenyl-1-picrylhydrazyl (DPPH) radicals (Figure 5, Table 3). It was found that the DPPH radicals scavenging activity of Au NPs is directly proportional to the amount of reductants used for their synthesis. The results showed the inhibition of DPPH free radicals after 15 min. Initially, both reductants showed a clear radical scavenging activity compared to Au NPs obtained on their basis. However, in both cases, after 3 days, the antioxidant activity of reductants and nanoparticles leveled out and reached about 30%. After this time, slightly higher antiradical activity was observed for the samples with the onion extract, which reversed after 6 days, where the samples with sodium citrate were the most effective.

### 2.4. Characterization of Hydrogel

**Figure 6:**
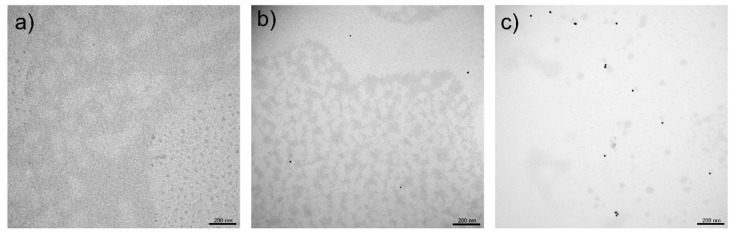
TEM images for hydrogel masks: (**a**) control and obtained using the reducer; (**b**) onion extract; (**c**) sodium citrate on a scale of 200 nm.

The analysis of hydrogel morphology was performed using microscopic methods. TEM images (Figure 6) were taken to check the distribution of nanoparticles in the hydrogel masks. In the case of hydrogels with nanoparticles (Figure 6b,c), we observe uniformly distributed nanostructures in the gel network.

**Figure 7:**
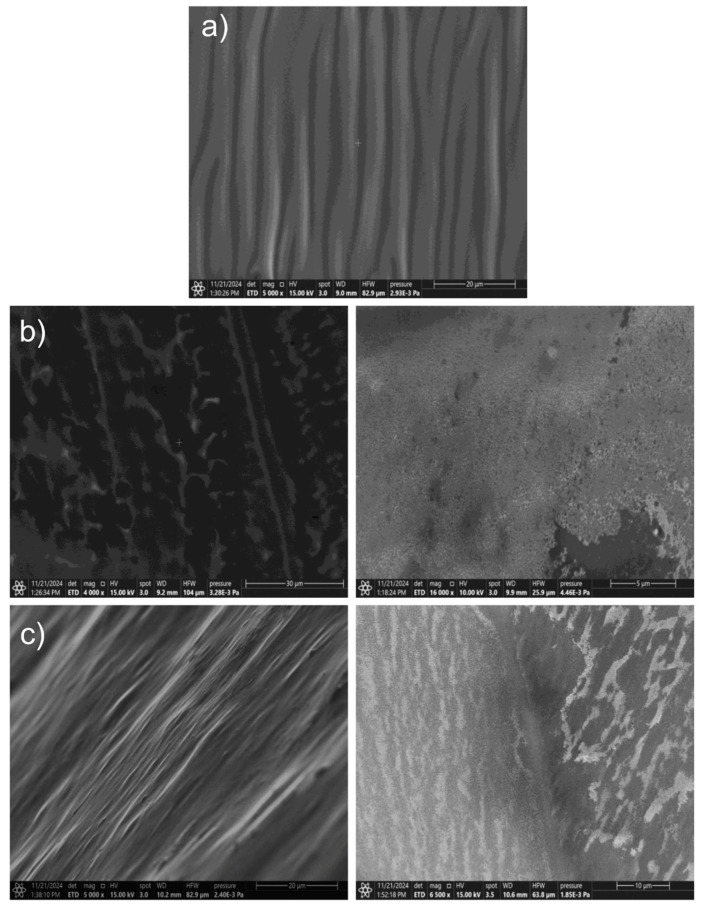
SEM images for hydrogel masks: (**a**) control and obtained using the reducer; (**b**) onion extract; (**c**) sodium citrate with smaller (**left**) and larger (**right**) zoom.

On the other hand, SEM images (Figure 7) showed that the unmodified HA-based hydrogel has a very uniform and smooth structure without visible porosities. In turn, Figure 7b,c show surface roughness and many spherical nanoparticles embedded in the hydrogel structure.

**Figure 8:**
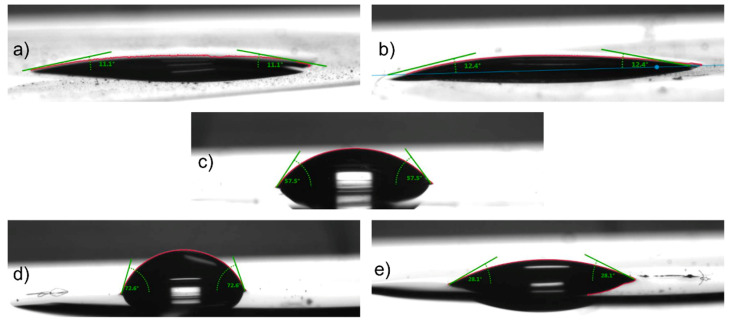
Water contact angle measurement results for: Au NPs obtained using the reducer (**a**) onion extract; (**b**) sodium citrate; (**c**) hydrogel mask (control, without NPs); (**d**) hydrogel mask Au NPs obtained using the onion extract; (**e**) hydrogel mask Au NPs obtained using the sodium citrate.

The outcomes from contact angle measurements demonstrate that selecting a reducing agent in the synthesis of Au NPs markedly affects the wettability of the resultant nanoparticles (Figure 8). The contact angle quantifies the wetting of a solid surface by a liquid, where smaller angles denote enhanced wettability, and larger angles signify diminished wettability.

The contact angles for the Au NPs are comparatively low, indicating that both onion extract and sodium citrate effectively generate hydrophilic nanoparticles. The minor variation in contact angles between the two reducing agents (11.1° for onion extract and 12.4° for sodium citrate) may be ascribed to the distinct functional groups and molecular architectures of the reducing agents, which can influence the surface characteristics of the Au NPs. As a biological reducing agent, onion extract probably alters the surface chemistry compared to inorganic sodium citrate, which may account for the noted difference in wettability.

Incorporating Au NPs into a hydrogel mask modifies the wettability of the hydrogel to align with the wettability characteristics of the nanoparticles. The control hydrogel mask, devoid of Au NPs, exhibits a contact angle of 57.5°, signifying a moderately hydrophilic surface. Incorporating Au NPs synthesized with onion extract results in an increased contact angle of 72.6°, indicating that the hydrogel exhibits enhanced hydrophobicity. This alteration may result from the onion extract-derived Au NPs conferring a more hydrophobic surface property to the hydrogel, likely attributable to organic residues from the onion extract on the nanoparticle surface.

In contrast, the addition of sodium citrate-synthesized Au NPs to the hydrogel mask results in a substantial reduction in the contact angle to 28.3°, signifying a marked increase in the hydrophilicity of the hydrogel. This outcome indicates that the sodium citrate-capped Au NPs possess a significant affinity for water, which they transfer to the hydrogel matrix. The notable reduction in contact angle relative to the control and onion extract—Au NPs may be attributed to the citrate ions on the nanoparticle surface enhancing water interaction and spreading on the hydrogel surface.

The selection of a reducing agent in the synthesis of Au NPs significantly influences the wettability of the nanoparticles and, subsequently, the wettability of composite materials such as hydrogel masks upon incorporation. The findings underscore the significance of choosing a suitable reducing agent to customize the surface characteristics of nanomaterials for particular applications, including creating hydrophilic or hydrophobic coatings, sensors, or biomedical devices.

### 2.5. Cosmetic Properties

**Figure 9:**
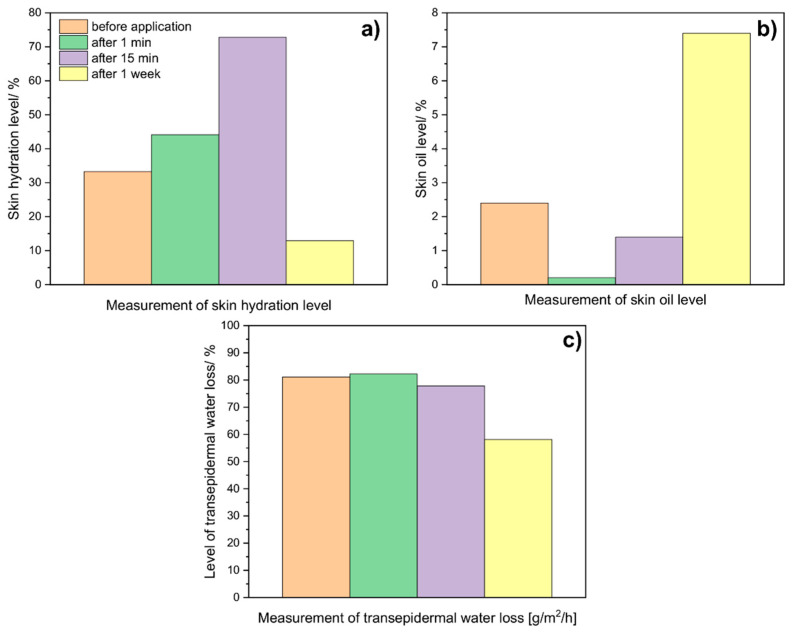
Average results of (**a**) skin hydration level, (**b**) oil level, and (**c**) transepidermal water loss (TEWL), were obtained for the skin before, after 1 min, 15 min, and 1 week after applying a hydrogel mask (control, without NPs).

The studies of the primary parameters of skin: hydration, sebum, TEWL, and skin robustness were conducted in a closed environment with a constant temperature (25 ± 2 °C) and humidity (25–30% RH); the results are presented in Figure 9 and Table 4.

As can be seen in the graphs below, skin hydration (Figure 9a) increases over time after the application of the hydrogel to the skin. The rapid decrease visible after a week can be attributed to the evaporation of most water molecules from the hydrogel and the skin. On the other hand, measurements taken to examine the level of skin oiliness (Figure 9b) showed that after application alone, the oil level drops to practically 0. After a week, the recorded level is the highest. The above results allow us to conclude that hydrogels may have sebo-normalizing properties. In turn, studies of transepidermal water loss (Figure 9c) showed that after the hydrogel application, this level is slightly higher than before the application itself. With time, a decrease in the value is noted; after a week, a level of about 58 was reported. The above results showed that hydrogels have a moisturizing and normalizing effect.

### 2.6. Cytotoxicity Results 

**Figure 10:**
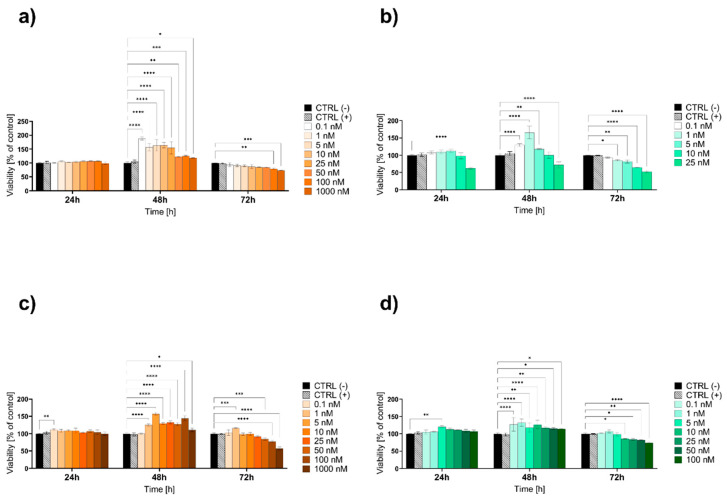
The viability of the HaCaT epidermal cell line evaluated by the MTT assay after 24, 48, and 72 h of exposure to lower concentrations of (**a**) hydrogel citrate Au NPs, (**b**) hydrogel onion Au NPs, (**c**) liquid citrate Au NPs, and (**d**) liquid onion Au NPs. Data are expressed as mean values ± SD from three independent experiments. * *p* < 0.05; ** *p* < 0.01; *** *p* < 0.001; **** *p* < 0.0001 versus negative control (CTRL−). The positive control (CTRL+) was water for the liquid formulations or hydrogel without Au NPs for the hydrogel formulations.

The study investigating the effects of citrate-based Au NPs in a hydrogel formulation revealed a dynamic, time-dependent cellular response (Figure 10a). At 24 h, there were no significant changes in cell viability across all nanoparticle concentrations. At 48 h, a notable increase in cell proliferation was observed at most concentrations, particularly between 0.1 and 25 nM (*p* < 0.0001), indicating a stimulatory effect. By 72 h, this proliferative effect had moderated, with cell viability stabilizing around 79.77% (*p* = 0.0067) and 73.61% (*p* = 0.0002) at the highest concentrations of 100 and 1000 nM, respectively, indicating a maintained yet reduced cellular viability at these higher concentrations (Figure 10a).

Similarly, the effects of onion-based Au NPs in a hydrogel formulation were assessed (Figure 10b). At 24 h, no significant changes in cell viability were observed, except for a decrease to 62.30% at 25 nM (*p* < 0.0001), while at 48 h, lower concentrations increased viability, but 25 nM resulted in a further decrease around 72.22% (*p* < 0.0001). By 72 h, viability was reduced to 64.67% at 10 nM and 52.47% at 25 nM (*p* < 0.0001) (Figure 10b).

At 24 h, the citrate-based Au NPs in liquid form showed a slight increase in cell viability at 0.1 nM (*p* = 0.0057), while no significant changes were observed at higher concentrations. By 48 h, there was a significant increase in cell viability across most concentrations, particularly from 1 to 100 nM (*p* < 0.0001), indicating a strong proliferative response. At 72 h, cell viability decreased to 84.66% at 50 nM (*p* = 0.0002), 77.36% at 100 nM (*p* < 0.0001), and 56.94% at 1000 nM (*p* < 0.0001), suggesting a concentration-dependent cytotoxic effect (Figure 10c).

For the liquid form, at 24 h, the onion-based Au NPs showed no significant changes in cell viability across most concentrations, except for a significant increase at 5 nM (*p* = 0.0012). By 48 h, there was a significant increase in cell viability across all tested concentrations, particularly at 0.1, 1 and 10 nM (*p* < 0.0001). At 72 h, the trend shifted, with cell viability decreasing to 83.87% at 25 nM, 82.12% at 50 nM, and 74.22% at 100 nM (*p* = 0.0147; *p* = 0.0054 and *p* < 0.0001, respectively) (Figure 10d).

**Figure 11:**
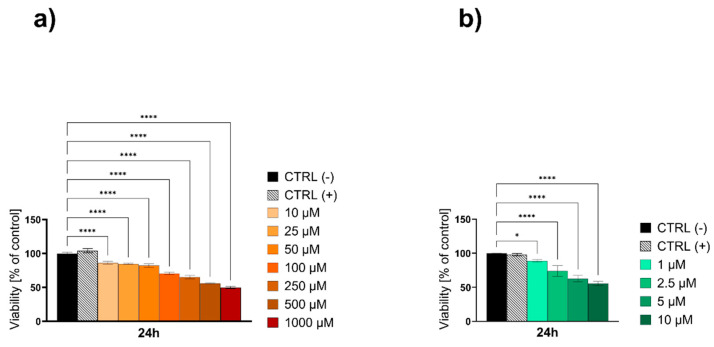
The viability of the HaCaT epidermal cell line evaluated by the MTT test after 24 h of exposure to high concentrations of (**a**) Au NPs citrate and (**b**) Au NPs onion. Data are expressed as mean values ± SD from three separate experiments. * *p* < 0.05; ** *p* < 0.01; **** *p* < 0.0001 versus negative control (CTRL−). The positive control (CTRL+) was water for the liquid formulations.

**Figure 12:**
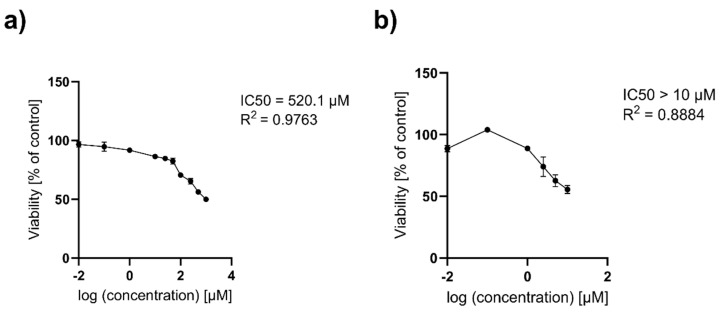
The IC_50_ values calculated by a nonlinear regression analysis for HaCaT (**a**) Au NPs citrate; (**b**) Au NPs onion after 24 h of exposure to high concentrations. Data are expressed as means ± SD for three separate experiments. R^2^—coefficient of determination.

To further explore the cytotoxic potential of the Au NPs and to precisely determine the IC_50_ values, higher concentrations ranging from 10 to 1000 µM for citrate (Figure 11a) and from 1 to 10 µM for onion-based formulations (Figure 11b) were tested over 24 h in their liquid forms. For the citrate-based Au NPs, as the concentration increased, the cytotoxic effect became more pronounced, with cell viability progressively declining to 86.28% at 10 µM, 82.39% at 50 µM, and 70.59% at 100 µM (*p* < 0.0001). This downward trend continued at even higher concentrations, with further decreases to 65.31% at 250 µM and 56.14% at 500 µM (*p* < 0.0001). The most substantial reduction was observed at 1000 µM, where cell viability declined to 49.98% (*p* < 0.0001), highlighting the strong cytotoxic response at these elevated concentrations. The IC_50_ value for the citrate-based Au NPs was determined using a variable slope (four parameters) model. The best-fit IC_50_ was calculated to be 520.1 µM, with a logIC_50_ of 2.716 (Figure 12a and Table 5). The results for onion-based Au NPs showed that at 1 µM concentration, there were modest but statistically significant reductions in cell viability, with viability decreasing to approximately 88.76% (*p* = 0.0115), respectively. At 2.5 µM, cell viability significantly decreased to 74.01% (*p* < 0.0001). This trend continued at 5 and 10 µM, where viability declined further to 62.66% and 55.61%, respectively (*p* < 0.0001). As the highest tested concentration (10 µM) did not reduce cell viability below 50%, the IC_50_ for the onion-based Au NPs is determined to be greater than 10 µM (Figure 12b and Table 5).

According to standard [44], cytotoxicity is categorized based on cell viability as follows: concentrations resulting in viability above 80% are considered non-cytotoxic, those between 80% and 60% represent weak cytotoxicity, between 60% and 40% moderate cytotoxicity, and below 40% strong cytotoxicity. In the case of hydrogel formulations, citrate-based Au NPs exhibit non-cytotoxicity up to 25 nM (Figure 10a), whereas onion-based demonstrate weak to moderate cytotoxicity at concentrations equal to or exceeding 10 nM (Figure 10b). For liquid formulations, citrate-based Au NPs are safe up to 50 nM (Figure 10c), while onion-based Au NPs remain non-cytotoxic up to 25 nM, with weak cytotoxicity observed at 50–100 nM (Figure 10d).

In summary, hydrogel formulations of both citrate-based and onion-based Au NPs show comparable cytotoxicity profiles. However, liquid formulations of citrate-based nanoparticles display a broader safety margin, remaining non-cytotoxic up to 50 nM, compared to onion-based nanoparticles, which begin to exhibit weak cytotoxicity at concentrations of 50 nM and above. This indicates that citrate-based nanoparticles in liquid formulations may be safer for biological applications. Additionally, liquid formulations generally exhibit lower toxicity compared to hydrogels, suggesting that the hydrogel matrix could enhance cellular exposure or nanoparticle uptake, potentially amplifying toxic effects.

### 2.7. Nanoparticles Permeability Tests

**Figure 13:**
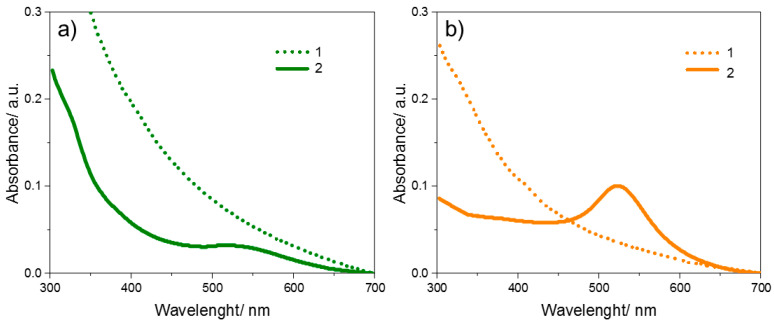
Absorption spectra for Au NPs obtained using the reducer: (**a**) onion extract; (**b**) sodium citrate. 1—for the receiving solution from the Franz diffusion chamber and 2—for the solution of nanoparticles used as an ingredient in the hydrogel mask (initial).

Permeability tests are extremely important to assess the ability of nanoparticles to penetrate the skin layers. This is essential for the development of advanced topical and transdermal delivery systems. These tests can determine whether such materials have the ability to be administered locally or systemically. UV–Vis Spectroscopy (Figure 13) was used to determine the permeation of the nanoparticles contained in the hydrogel formulations. The absorbance maximum for the Au NPs obtained was in the region of 530 nm. The biological model membrane was pig skin from the hind femur. The limit of quantification of the nanoparticle concentration was 5 × 10^−8^ M. It was shown that in hydrogel masks containing both nanoparticles from onion extract and sodium citrate, the nanoparticles did not pass through the selected skin model.

## 3. Materials and Methods

### 3.1. Materials and Reagents

Tetrachloroauric acid (HAuCl_4_), sodium borohydride (NaBH_4_), sodium citrate 2-aqueous (C_6_H_5_Na_3_O_7_·2H_2_O), triethylamine (TEA), and 2,2-diphenyl-1-picrylhydrazyl radical (DPPH) were purchased from Sigma-Aldrich (Poznań, Poland). High molecular weight hyaluronic acid (HA) with a molecular weight in the range of 0.85–1.15 MDa was purchased from Chemat (Los Angeles, CA, USA). Deionized water used for syntheses, preparation of hydrogel masks, and analyses came from the Hydrolab system (Straszyn, Poland) installed in our laboratory.

### 3.2. Synthesis

#### 3.2.1. Preparation of Onion Extract

First, the onion was peeled, cut into smaller pieces, and weighed (20 g) to obtain the onion extract. Then, it was transferred to a mortar, where it was crushed. The crushed onion, together with the juice, was transferred to a beaker, and about 40 mL of water was added and boiled for approximately half an hour. The last step was to filter the extract.

#### 3.2.2. Synthesis of Au NPs

For the synthesis of Au NPs, 3 mL of a 5 mM tetrachloroauric acid solution was mixed with 50 mL of water. This solution was then stirred with a magnetic stirrer (IKA^®^, model RT 5, Staufen, Germany) at 120 °C. It was heated until it reached boiling point, and then 3 mL of reducing agent was added. As the latter mentioned, an onion extract or sodium citrate (0.02 M) was used. When the solution obtained a ruby color, it was heated for about 5 more minutes. Then, the purification process was carried out by centrifuging the colloid, removing the supernatant, and dispersing it in deionized water. The process was performed in three repetitions, and finally, the nanoparticles were suspended in water.

#### 3.2.3. Synthesis of Hydrogel Masks

**Scheme 1:**
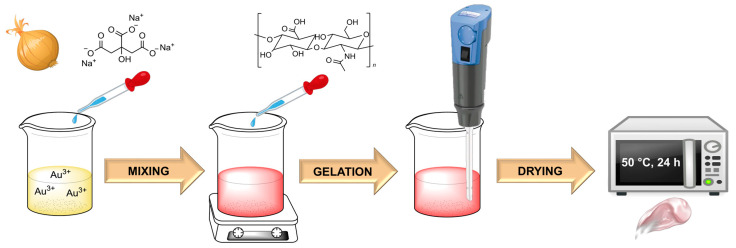
Scheme for obtaining HA-based hydrogels with Au NPs.

The following ingredients were used to prepare the hydrogels for all the nanoparticles obtained: 0.01 g of HA was added to 3 mL of H_2_O containing 0.1 mL of Au NPs colloids. For the control mask, no nanoparticles were added. The solution was dispersed for 20 min using a homogenizer from IKA^®^, model T10 Basic ULTRA-TURRAX^®^. After this time, 3 drops each of triethanolamine were added and mixed until a uniform gel-like consistency was obtained. The masks obtained in this way were dried in an oven at 50 °C, for 24 h. The whole procedure for obtaining hyaluronic acid-based hydrogels loaded with Au NPs is shown in Scheme 1.

### 3.3. Methods

#### 3.3.1. Characterization

Phytochemical analyses of onion extract were performed on an HPLC-MS system consisting of an Agilent Technologies liquid chromatograph (model 1200) (Santa Clara, CA, USA) and a Bruker Daltonics HCT Ultra spectrometer (Billerica, MA, USA). Phenolic acids and flavonols were analyzed using a Gemini C18 column (150 × 4.6 mm 5 µm, Phenomenex, Torrance, CA, USA). The mobile phase consisted of 0.1% HCOOH (*v*/*v*) in (A) water and (B) ACN, the column temperature was 25 °C and the flow rate was 0.5 mL/min. The gradient elution was modified as follows: 0–5 min 10% B, 5–35 min from 10% to 60% B, 35–40 min from 60% to 90% B, 40–55 min from 90 to 10% B. The posttermination time was 15 min. The injection volume was 20 μL. Chromatograms were obtained at 254 nm. The following instrument conditions were used during the mass analysis: drying gas flow 30 psi, 10 L/min, capillary temperature 350 °C, capillary voltage: ±4 kV. The mass spectrum was measured in the range 50–1000 m/z target mass at 500 m/z, fullscan analysis in positive and negative polarization. UV–Vis spectra were recorded using a Perkin Elmer UV–Vis spectrophotometer, model Lambda 650 (Shelton, CT, USA), in the 200–800 nm range. Fourier transform infrared (FT-IR) spectra, on the other hand, were performed with a Bruker IFS66 FT-IR spectrometer (Ettlingen, Germany) using the KBr pellet method. Each FT-IR spectrum was recorded in the range of 4000 to 1000 cm^−1^. The zeta potential was measured using the Electrophoretic Light Scattering (ELS) method. The size of nanoparticles was determined using a dynamic light scattering (DLS) technique in the measurement range from 0.3 nm to 10 μm (particle diameter). Both measurements were carried out on a Litesizer 500, ANTON-PAAR instrument (Graz, Austria). Transmission Electron Microscope (TEM) images were taken with a Tecnai G2 Spirit BioTWIN FEI microscope (Eindhoven, The Netherlands). Scanning Electron Microscope (SEM) images were taken with a Prisma E Thermofisher Scientific microscope (Waltham, MA, USA). Analysis of skin parameters was carried out using the MPA 6 Multi Probe Adapter Corneometer^®^ CM 82 modular device, Sebumeter^®^ SM 815, and Tewameter^®^ TM 300. Wettability measurements were carried out using a Krüss Drop Shape Analyzer (DSA100) goniometer (Hamburg, Germany). The contact angle technique involves the measurement of the contact angles of a drop of distilled water on the surface of a tested sample. At first, the structure was dissolved in ethanol, dispersed by ultrasound, spotted onto a glass slide, and allowed to dry. A 6 µL drop of water was deposited using a syringe onto a sample placed on a glass slide. The image of the drop was recorded with a CCD camera. Measurements were repeated 20 times, and the mean value was given with standard deviation [45,46,47,48].

#### 3.3.2. Antioxidant Assay

The DPPH test was used to check the antioxidant activity. This is a simple and rapid method for testing total antioxidant scavenging activity. This publication uses the approach proposed by S. Menon et al. [35]. In view of this, 4.3 mg of the DPPH compound was dissolved in 3.3 mL of methanol solution, which was covered with aluminum foil to protect it from light. For the control, 150 μL of the prepared DPPH solution was taken, dissolved into 3 mL of methanol, and the absorbance was measured at 517 nm. To measure a sample of Au NPs obtained with different reductants, 0.1 mL of them were taken. In contrast, for the reductant itself, amounts were added to synthesize the portion of the nanoparticles. Then, 150 μL of DPPH solution was added to each sample and diluted with methanol to 3 mL. The absorbance was checked after 15 min at the same wavelength, setting methanol as a blank solution. Measurements were taken again after 1 h, 1, 2, 3, 6, and 7 days. DPPH scavenging activity was calculated using the formula:

% antioxidant potential = 100 (A_0_ − A_s_)/A_0_

A_0_—absorbance of control.
A_s_—absorbance of the sample.

#### 3.3.3. Cosmetic Tests of Hydrogel

The study used pig skin from the animal’s hind legs. A portion of the hydrogel was applied to the skin, and tests were performed. The MPA 6 Multi Probe Adapter was used to assess skin condition. Skin hydration was measured using the Corneometer^®^ CM 825, the Sebumeter^®^ SM 815 was used to measure sebum on the skin surface, and the Tewameter^®^ TM Hex was used to measure transepidermal water loss (TEWL).

#### 3.3.4. Cytotoxicity Studies

The viability of HaCaT keratinocytes (Cytion, Eppelheim, Germany) was quantitatively evaluated using the MTT assay, a colorimetric method based on the reduction of the yellow tetrazolium salt, 3-(4,5-dimethylthiazol-2-yl)-2,5-diphenyltetrazolium bromide (Sigma-Aldrich, Saint Louis, MO, USA), into insoluble purple formazan crystals by metabolically active cells. Cells were seeded in 96-well microplates at a density of 12–14 × 10^3^ cells per 100 µL per well. Following a 24 h incubation period, the cells were treated with varying concentrations gold-based nanostructures, freshly prepared in a serum-free culture Dulbecco’s Modified Eagle Medium (DMEM, Capricorn Scientific GmbH, Ebsdorfergrund, Germany). After a further 24 h exposure period, MTT reagent was added to each well. The reaction was allowed to proceed for 3 h, after which the medium was carefully removed, and the resultant formazan crystals were solubilized in dimethyl sulfoxide (DMSO, Sigma-Aldrich, Saint Louis, MO, USA). Absorbance was measured at 570 nm using a Synergy™ HT microplate reader (Charlotte, VT, USA), with the data normalized to the negative control group (untreated cells), which was set to 100%. For the positive control, water was used for liquid formulations, while a hydrogel without nanoparticles served as the control for hydrogel formulations. The experimental results are expressed as the mean ± SD, based on at least three independent experiments, each performed in triplicate. Data were analyzed using GraphPad Prism 8.0 software (Boston, MA, USA), employing one-way ANOVA followed by Tukey’s post hoc test. Statistical significance was defined as *p* values < 0.05. The IC_50_ values were calculated using nonlinear regression analysis with the log(inhibitor) vs. normalized response model.

#### 3.3.5. Ex Vivo Diffusion System

The study used porcine skin from the hind leg of the animal. Full-thick skin was prepared, fat was removed, and hair was shaved. All pieces of porcine skin were then stored in a freezer at −25 °C. According to Franz [49], this storage method does not damage the skin, and no difference in permeability was observed between fresh and frozen skin pieces. To characterize the penetration of Au NPs, as stated in the introduction, almost all published works on ex vivo release from semisolids have used vertical Franz-type cells. The Franz-type diffusion cell used in this study was made of borosilicate glass with a 2 cm diameter hole in the center, the same size as the hole in the vertical receptor cell, which is placed on top of the membrane. The membrane was placed on top of the vertical receptor cell and tightly clamped. The exposed skin area was 3.1 cm^2^, and the average thickness of the membranes was 1.7 mm. The receptor cell was filled with buffer (pH 7.4), and a small Teflon-coated magnetic stirrer was used for stirring at 500 rpm. The flow rate was 5 mL/min during the entire study at a constant temperature of 32 ± 0.5 °C. After reaching equilibrium for 30 min, the appropriate Au NP hydrogel sample was applied to the skin surface. Then, the solution from the cell was examined for the presence of nanoparticles using UV–Vis Spectroscopy.

## 4. Conclusions

In conclusion, integrating green synthesis methods with nanostructure designs could lead to the development of the synthesis of new nanoparticles or nanomaterials with broader applicability. This study introduces a novel approach for synthesizing Au NPs with onion *Allium cepa* L. extract as a reductant using a green chemistry method. Comprehensive characterization using various analytical techniques revealed Au NPs with an average 10–30 nm diameter. Zeta potential measurements showed that all samples were stable, with an average zeta potential equal to –34.9 mV. The cytotoxic results for onion-based Au NPs showed that the highest tested concentration (10 µM) did not reduce cell viability below 50%; the IC_50_ for the onion-based Au NPs is determined to be greater than 10 µM. The results showed the inhibition of DPPH free radicals after 15 min. In addition, onion-based Au NPs have similar parameters to those obtained using sodium citrate as a reducer. They have the advantage of being environmentally friendly raw materials, which opens up new possibilities for using them in biomedicine, cosmetology, and environmental sciences. However, an interesting difference is the result of wettability studies, which show that the use of standard reducers for the synthesis of nanoparticles, with which the gel is later modified, causes a more desirable surface character in possible medical or cosmetic applications.

In the second step, we successfully prepared HA-based hydrogel masks with onion-based Au NPs. The results of studies of the primary parameters of skin: hydration, sebum, TEWL, and skin robustness show that hydrogels have a moisturizing and normalizing effect. Moreover, permeability tests showed that the nanoparticles remained on the skin surface without penetrating the transepidermal barrier (pig skin as a human skin model).

The results of our studies delivered a solid foundation for future innovations in nanoparticle synthesis and their applications as safe ingredients in cosmetics products.

## Figures and Tables

**Table 1:** Results of the measurement of particle size distribution.

Au NPs Obtained Using the Reducer	Average Size (SD) [nm]	Standard Deviation [nm]	Area [%]	Polydispersity Index (PdI)
onion extract	24.02	8.68	60.92	0.23
	198.97	94.88	37.07	
sodium citrate	66.97	24.22	92.72	0.08

**Table 2:** The mean zeta potential results of the samples of Au NPs obtained using the reducer onion extract and sodium citrate.

Au NPs Obtained Using the Reducer	Mean Zeta Potential (SD) [mV]	Deviation [mV]
onion extract	−34.9	0.4
sodium citrate	−23.0	0.5

**Table 3:** The obtained results of the antioxidant potential expressed as a percentage by the DPPH method for the reductants and nanoparticles obtained with their use.

Time	Antioxidant Potential [%]			
	Onion Extract	Au NPs with Onion Extract	Sodium Citrate	Au NPs with Sodium Citrate
15 min	7.08	0.94	7.53	1.15
1 h	9.02	2.45	8.92	2.09
1 day	21.03	17.51	22.56	14.65
2 days	25.9	23.04	27.78	18.9
3 days	32.94	35.76	28.6	29.33
6 days	39.93	38.91	39.97	41.37
7 days	41.57	39.87	47.95	45.13

**Table 4:** Average results were obtained for the skin before, after 1 min, 15 min, and 1 week after applying a hydrogel mask (control, without NPs).

	Hydration Avg	Sebum Values	Avg TEWL Robust [g/m^2^/h]	Avg RH Skin Robust [%]
Before application	33.28	2.4	81.13	109.91
After 1 min	44.12	0.2	82.25	110.17
After 15 min	72.82	1.4	77.87	107.70
After 1 week	12.96	7.4	58.13	94.42

**Table 5:** IC_50_ values for HaCaT cells exposed to Au NPs citrate and Au NPs onion.

	HaCaT	
	Au NPs Citrate	Au NPs Onion
IC_50_	520.1 µM (102.44 ppm)	>10 µM (>1.9697 ppm)
logIC_50_	2.716087	-

## Data Availability

Data are contained within the article.

## References

[1] Mishra R.K., Tiwari S.K., Mohapatra S., Thomas S. (2019). Efficient Nanocarriers for Drug-Delivery Systems. Nanocarriers for Drug Delivery.

[2] Kowalska A., Adamska E., Grobelna B. (2024). Medical Applications of Silver and Gold Nanoparticles and *Core-Shell* Nanostructures Based on Silver or Gold Core: Recent Progress and Innovations. ChemMedChem.

[3] Rizzi V., Gubitosa J., Fini P., Nuzzo S., Agostiano A., Cosma P. (2021). Snail Slime-Based Gold Nanoparticles: An Interesting Potential Ingredient in Cosmetics as an Antioxidant, Sunscreen, and Tyrosinase Inhibitor. J. Photochem. Photobiol. B Biol..

[4] Majerič P., Jović Z., Švarc T., Jelen Ž., Horvat A., Koruga D., Rudolf R. (2023). Physicochemical Properties of Gold Nanoparticles for Skin Care Creams. Materials.

[5] Gulati S., Singh P., Diwan A., Mongia A., Kumar S. (2020). Functionalized Gold Nanoparticles: Promising and Efficient Diagnostic and Therapeutic Tools for HIV/AIDS. RSC Med. Chem..

[6] Jha C.B., Singh C., Randhawa J.K., Kaul A., Varshney R., Singh S., Kaushik A., Manna K., Mathur R. (2024). Synthesis and Evaluation of Curcumin Reduced and Capped Gold Nanoparticles as a Green Diagnostic Probe with Therapeutic Potential. Colloids Surf. B Biointerfaces.

[7] Puzari U., Khan M.R., Mukherjee A.K. (2024). Development of a Gold Nanoparticle-Based Novel Diagnostic Prototype for in Vivo Detection of Indian Red Scorpion (*Mesobuthus tamulus*) Venom. Toxicon X.

[8] Theodosiou M., Chalmpes N., Gournis D., Sakellis E., Boukos N., Kostakis M., Thomaidis N.S., Efthimiadou E.K. (2024). Amino Acid Driven Synthesis of Gold Nanoparticles: A Comparative Study on Their Biocompatibility. Mater. Chem. Phys..

[9] Ibrahim B., Akere T.H., Chakraborty S., Valsami-Jones E., Ali-Boucetta H. (2023). Functionalized Gold Nanoparticles Suppress the Proliferation of Human Lung Alveolar Adenocarcinoma Cells by Deubiquitinating Enzymes Inhibition. ACS Omega.

[10] Musielak M., Boś-Liedke A., Piwocka O., Kowalska K., Markiewicz R., Szymkowiak B., Bakun P., Suchorska W.M. (2023). The Role of Functionalization and Size of Gold Nanoparticles in the Response of MCF-7 Breast Cancer Cells to Ionizing Radiation Comparing 2D and 3D In Vitro Models. Pharmaceutics.

[11] Lorenzana-Vázquez G., Pavel I., Meléndez E. (2023). Gold Nanoparticles Functionalized with 2-Thiouracil for Antiproliferative and Photothermal Therapies in Breast Cancer Cells. Molecules.

[12] Kong F.-Y., Zhang J.-W., Li R.-F., Wang Z.-X., Wang W.-J., Wang W. (2017). Unique Roles of Gold Nanoparticles in Drug Delivery, Targeting and Imaging Applications. Molecules.

[13] Huang H., Liu R., Yang J., Dai J., Fan S., Pi J., Wei Y., Guo X. (2023). Gold Nanoparticles: Construction for Drug Delivery and Application in Cancer Immunotherapy. Pharmaceutics.

[14] Rai M., Singh S.K., Singh A.K., Prasad R., Koch B., Mishra K., Rai S.B. (2015). Enhanced Red Upconversion Emission, Magnetoluminescent Behavior, and Bioimaging Application of NaSc_0.75_Er_0.02_Yb_0.18_Gd_0.05_F_4_@AuNPs Nanoparticles. ACS Appl. Mater. Interfaces.

[15] Batool M., Fatima B., Hussain D., Mahmood R., Imran M., Akhter S., Khan M.S., Majeed S., Najam-ul-Haq M. (2024). Radiotracer Labelled Thymohydroquinyl Gallate Capped Gold Nanoparticles as Theranostic Radiopharmaceutical for Targeted Antineoplastic and Bioimaging. J. Pharm. Anal..

[16] Pajović J.D., Dojčilović R.J., Kaščáková S., Réfrégiers M., Božanić D.K., Djoković V. (2023). Enhanced Resonance Energy Transfer in Gold Nanoparticles Bifunctionalized by Tryptophan and Riboflavin and Its Application in Fluorescence Bioimaging. Colloids Surf. B Biointerfaces.

[17] Chalmpes N., Tantis I., Alsmaeil A.W., Bourlinos A.B., Giannelis E.P. (2024). Design, Synthesis, and Evaluation of Noble Metal Nanoparticles and In Situ-Decorated Carbon-Supported Nanoparticle Electrocatalysts Using Hypergolic Reactions. Chem. Mater..

[18] Lee K.J., Nallathamby P.D., Browning L.M., Osgood C.J., Xu X.-H.N. (2007). In Vivo Imaging of Transport and Biocompatibility of Single Silver Nanoparticles in Early Development of Zebrafish Embryos. ACS Nano.

[19] Sakore P., Bhattacharya S., Belemkar S., Prajapati B.G., Elossaily G.M. (2024). The Theranostic Potential of Green Nanotechnology-Enabled Gold Nanoparticles in Cancer: A Paradigm Shift on Diagnosis and Treatment Approaches. Results Chem..

[20] Zuhrotun A., Oktaviani D.J., Hasanah A.N. (2023). Biosynthesis of Gold and Silver Nanoparticles Using Phytochemical Compounds. Molecules.

[21] Rao N.V., Rho J.G., Um W., Ek P.K., Nguyen V.Q., Oh B.H., Kim W., Park J.H. (2020). Hyaluronic Acid Nanoparticles as Nanomedicine for Treatment of Inflammatory Diseases. Pharmaceutics.

[22] Larrañeta E., Henry M., Irwin N.J., Trotter J., Perminova A.A., Donnelly R.F. (2018). Synthesis and Characterization of Hyaluronic Acid Hydrogels Crosslinked Using a Solvent-Free Process for Potential Biomedical Applications. Carbohydr. Polym..

[23] Lee W.H., Rho J.G., Yang Y., Lee S., Kweon S., Kim H.-M., Yoon J., Choi H., Lee E., Kim S.H. (2022). Hyaluronic Acid Nanoparticles as a Topical Agent for Treating Psoriasis. ACS Nano.

[24] Hwang H.S., Lee C.-S. (2023). Recent Progress in Hyaluronic-Acid-Based Hydrogels for Bone Tissue Engineering. Gels.

[25] Lin X., Fu T., Lei Y., Xu J., Wang S., He F., Xie Z., Zhang L. (2023). An Injectable and Light Curable Hyaluronic Acid Composite Gel with Anti-Biofilm, Anti-Inflammatory and pro-Healing Characteristics for Accelerating Infected Wound Healing. Int. J. Biol. Macromol..

[26] Niu J., Yuan M., Liu Y., Wang L., Tang Z., Wang Y., Qi Y., Zhang Y., Ya H., Fan Y. (2022). Silk Peptide-Hyaluronic Acid Based Nanogels for the Enhancement of the Topical Administration of Curcumin. Front. Chem..

[27] Kuche K., Pandey P.K., Patharkar A., Maheshwari R., Tekade R.K. (2019). Hyaluronic Acid as an Emerging Technology Platform for Silencing RNA Delivery. Biomaterials and Bionanotechnology.

[28] Varela-Aramburu S., Su L., Mosquera J., Morgese G., Schoenmakers S.M.C., Cardinaels R., Palmans A.R.A., Meijer E.W. (2021). Introducing Hyaluronic Acid into Supramolecular Polymers and Hydrogels. Biomacromolecules.

[29] Tavsanli B., Okay O. (2017). Mechanically Strong Hyaluronic Acid Hydrogels with an Interpenetrating Network Structure. Eur. Polym. J..

[30] Elvitigala K.C.M.L., Mubarok W., Sakai S. (2023). Tuning the Crosslinking and Degradation of Hyaluronic Acid/Gelatin Hydrogels Using Hydrogen Peroxide for Muscle Cell Sheet Fabrication. Soft Matter.

[31] Raza H., Ashraf A., Shamim R., Manzoor S., Sohail Y., Khan M.I., Raza N., Shakeel N., Gill K.A., El-Marghany A. (2023). Synthesis and Characterization of Hyaluronic Acid (HA) Modified Polymeric Composite for Effective Treatment of Wound Healing by Transdermal Drug Delivery System (TDDS). Sci. Rep..

[32] Pramanik N., Ranganathan S., Rao S., Suneet K., Jain S., Rangarajan A., Jhunjhunwala S. (2019). A Composite of Hyaluronic Acid-Modified Graphene Oxide and Iron Oxide Nanoparticles for Targeted Drug Delivery and Magnetothermal Therapy. ACS Omega.

[33] Mendes C., Thirupathi A., Zaccaron R.P., Corrêa M.E.A.B., Bittencourt J.V.S., Casagrande L.D.R., De Lima A.C.S., De Oliveira L.L., De Andrade T.A.M., Gu Y. (2022). Microcurrent and Gold Nanoparticles Combined with Hyaluronic Acid Accelerates Wound Healing. Antioxidants.

[34] Carvalho T., Bártolo R., Pedro S.N., Valente B.F.A., Pinto R.J.B., Vilela C., Shahbazi M.-A., Santos H.A., Freire C.S.R. (2023). Injectable Nanocomposite Hydrogels of Gelatin-Hyaluronic Acid Reinforced with Hybrid Lysozyme Nanofibrils-Gold Nanoparticles for the Regeneration of Damaged Myocardium. ACS Appl. Mater. Interfaces.

[35] Tohidi H., Maleki N., Simchi A. (2024). Conductive, Injectable, and Self-Healing Collagen-Hyaluronic Acid Hydrogels Loaded with Bacterial Cellulose and Gold Nanoparticles for Heart Tissue Engineering. Int. J. Biol. Macromol..

[36] Umer M., Nisa M.U., Ahmad N., Rahim M.A., Kasankala L.M. (2024). Quantification of Quercetin from Red Onion (*Allium cepa* L.) Powder via High-performance Liquid Chromatography-Ultraviolet (HPLC-UV) and Its Effect on Hyperuricemia in Male Healthy Wistar Albino Rats. Food Sci. Nutr..

[37] Puri C., Pucciarini L., Tiecco M., Brighenti V., Volpi C., Gargaro M., Germani R., Pellati F., Sardella R., Clementi C. (2020). Use of a Zwitterionic Surfactant to Improve the Biofunctional Properties of Wool Dyed with an Onion (*Allium cepa* L.) Skin Extract. Antioxidants.

[38] Osmani R. (2014). Nanosponges: The Spanking Accession in Drug Delivery—An Updated Comprehensive Review. Der Pharmacia Sinica.

[39] Godipurge S.S., Yallappa S., Biradar N.J., Biradar J.S., Dhananjaya B.L., Hegde G., Jagadish K., Hegde G. (2016). A Facile and Green Strategy for the Synthesis of Au, Ag and Au–Ag Alloy Nanoparticles Using Aerial Parts of *R. Hypocrateriformis* Extract and Their Biological Evaluation. Enzym. Microb. Technol..

[40] Mohan J.C., Praveen G., Chennazhi K.P., Jayakumar R., Nair S.V. (2013). Functionalised Gold Nanoparticles for Selective Induction of in Vitro Apoptosis among Human Cancer Cell Lines. J. Exp. Nanosci..

[41] Abboud Y., Eddahbi A., El Bouari A., Aitenneite H., Brouzi K., Mouslim J. (2013). Microwave-Assisted Approach for Rapid and Green Phytosynthesis of Silver Nanoparticles Using Aqueous Onion (*Allium cepa*) Extract and Their Antibacterial Activity. J. Nanostruct. Chem..

[42] Im M.H., Park Y.-S., Leontowicz H., Leontowicz M., Namiesnik J., Ham K.-S., Kang S.-G., Najman K., Gorinstein S. (2011). The Thermostability, Bioactive Compounds and Antioxidant Activity of Some Vegetables Subjected to Different Durations of Boiling: Investigation *in Vitro*. LWT-Food Sci. Technol..

[43] Adu R.E.Y. (2022). Dye Sensitized Solar Cell (DSSC) Fabrication Using Methanol Extract of Onion Peel as a Natural Sensitizer. J. Turk. Chem. Soc. Sect. A Chem..

[44] (2009). Biological Evaluation of Medical Devices—Part 5: Tests for in Vitro Cytotoxicity.

[45] Janik M., Głowacki M.J., Sawczak M., Wcisło A., Niedziałkowski P., Jurak K., Ficek M., Bogdanowicz R. (2022). Poly-l-Lysine-Functionalized Fluorescent Diamond Particles: pH Triggered Fluorescence Enhancement via Surface Charge Modulation. MRS Bull..

[46] Głowacki M.J., Ficek M., Sawczak M., Wcisło A., Bogdanowicz R. (2022). Fluorescence of Nanodiamond Cocktails: pH-Induced Effects through Interactions with Comestible Liquids. Food Chem..

[47] Adamska E., Niska K., Wcisło A., Grobelna B. (2021). Characterization and Cytotoxicity Comparison of Silver- and Silica-Based Nanostructures. Materials.

[48] Szczepańska E., Synak A., Bojarski P., Niedziałkowski P., Wcisło A., Ossowski T., Grobelna B. (2020). Dansyl-Labelled Ag@SiO_2_ Core-Shell Nanostructures—Synthesis, Characterization, and Metal-Enhanced Fluorescence. Materials.

[49] Franz T.J. (1975). Percutaneous Absorption on the Relevance of in Vitro Data. J. Investig. Dermatol..

